# Familial aggregation of lung cancer in a high incidence area in China

**DOI:** 10.1038/sj.bjc.6602465

**Published:** 2005-03-08

**Authors:** Y T Jin, Y C Xu, R D Yang, C F Huang, C W Xu, X Z He

**Affiliations:** 1 School of Public Health, Anhui Medical University, Hefei 230032, Anhui Province, China; 2 Yunnan Province Anti-epidemic Station, Kunming 650003, Yunnan Province, China; 3 Xuan Wei Anti-epidemic Station and Tumor Prevention and treatment Office, Xuanwei 655400, Yunnan Province, China; 4 Institute of Environmental Health and Engineering, Chinese Academy of Preventive Medicine, Beijing 100050, China

**Keywords:** family, proband, lung neoplasms, smoky coal exposure, smoking

## Abstract

To investigate whether lung cancer clusters in families in a high incidence county of China, an analysis was conducted using data on domestic fuel history and tobacco use for family members of 740 deceased lung cancer probands and 740 controls (probands' spouses). Lung cancer prevalence was compared among first-degree relatives of probands and of controls, taking into account various factors using logistic regression and generalised estimating equations. First-degree relatives of probands, compared with those of controls, showed an excess risk of lung cancer (odds ratio (OR)=2.05, 95% confidence interval (CI): 1.68–2.53). Overall, female relatives of probands had a greater risk than did their male counterparts, and the risk was 2.90-fold for parents of probands as compared with parents of spouses. Female relatives of probands had 2.67-fold greater risk than female controls. Lung cancer risk was particularly marked among mothers (OR=3.78, 95% CI: 2.03–7.12). Having two or more affected relatives was associated with a 2.69–5.40-fold risk increase. The risk elevation was also found for other cancers overall. Results confirm previous findings of a genetic predisposition to lung cancer, and also imply that lung cancer may share a genetic background with other cancers.

Lung cancer has been the leading cause of cancer death in China, but is particularly high in Xuan Wei County, Yunnan Province, China. In this rural county (population about 1.2 million), more than 95% of people are farmers. Tobacco smoking is common in males (40% or more), but rare in females (less than 0.1%). Lung cancer mortality is five times the national average and among the highest in China, yet females despite almost all being nonsmokers have the highest rate in China (eight times the national female average). It is unusual to find similar male and female lung cancer mortality rates (27.7 and 25.3 per 100 000, respectively) as in Xuan Wei County (Mumford *et al*, 1987).

Cigarette smoking has long been established as the predominant risk factor for lung cancer (Doll and Peto, 1978; IARC, 1986). Although an aetiological link between lung cancer mortality and domestic smoky coal use (polycyclic aromatic hydrocarbons) has been shown, the causes of lung cancer in Xuan Wei County have remained unexplained (Mumford *et al*, 1987; He *et al*, 1991). Host susceptibility factors, however, have not been explored there. Several studies have reported a slight increase of risk for relatives of lung cancer cases (Tokuhata and Lilienfeld, 1963; Mulvihill, 1976; Ooi *et al*, 1986; Bromen *et al*, 2000). Some of these, however, were limited to special groups such as nonsmokers or women, and this may have contributed to the different risks obtained (Schwartz *et al*, 1996; Wu *et al*, 1996; Brownson *et al*, 1997; Kreuzer *et al*, 1998; Pools *et al*, 1999). We have used improved modelling techniques to test the hypothesis that in Xuan Wei County, lung cancer cases are more likely than controls to have an affected relative.

## MATERIALS AND METHODS

### Study population

Probands, selected from the death records of the Office of Prevention and Treatment of Tumour in Xuan Wei County, had died of lung cancer during an 8-year period (1992–1999) in three communes (Cheng Guan, Lai Bin and Rong Cheng), which were ranked in the highest for lung cancer mortality (over 80 per 100 000) during 1973–1979. The residents of these communes were almost all farmers and had used smoky coal as their main fuel for cooking and heating, and had lived there for more than 20 years.

The controls were the probands' spouses without lung cancer who, because of the cultural characteristics of the target population, were assumed to have a similar environment and similar socioeconomic status. The controls were also farmers who would have lived over 20 years in Xuan Wei County. The first-degree relatives (parents and full siblings) of cases and controls were then identified (see below).

### Data collection

Lung cancer was defined as a primary cancer of the trachea, bronchus or lung (international classification of diseases (ICD)^#^ codes 162.0–162.9, 9th revision). Standard demographic characteristics of the probands and the identities of some of their next of kin were abstracted from death records. Trained interviewers used a standardised questionnaire to obtain information by face-to-face interviews from (in order of preference) spouse, parent or sibling. This information included the total tonnage of smoky coal or the number of tractor loads (which can be equated with tonnage) that were purchased annually; any change in the rate of their consumption of smoky coal; active and passive smoking exposure; nutritional details, medical history of participants and their families, and sociodemographic characteristerics.

For a 20% random sample, reliability of interview data was tested during the initial 6 months of the study by comparing responses between two members of the family. Cancer histories were verified by two methods: (1) a review of death certificates on a sample of relatives of probands and spouses who died in Xuan Wei County (80.4 and 70.4%, respectively) and (2) corroborative information from additional family contacts. The cancers were not restricted to a fixed time period, except that cases were excluded if diagnosed after data were collected.

All study subjects signed a consent form according to the guidelines of the World Medical Association Declaration of Helsinki.

### Statistical analysis

A dichotomous variable was created to code the history of lung cancer for each relative (parent, sibling). We determined stratified odds ratios (OR) separately for paternal and maternal lung cancer after calculating the frequencies of lung cancer in the various types of relatives. The potential confounders, age, region of residence and sex, were considered both in the design, by individual matching of cases and controls, and in the analysis, by applying univariate and multiple conditional logistic regression using the PHREG procedure in SAS software (SAS Institute Inc., 1997).

A measure of cumulative exposure to smoky coal use for a given individual was obtained by multiplying the annual rate of smoky coal use by the number of years. Coal consumption was generally constant for the households over the life cycle of the family. Three exposure categories were formed: >0–70, 70–140 and >140 tons. For cigarette smoking, pack-years (defined as cigarette packs smoked daily multiplied by years of smoking, with gram equivalents of leaf tobacco, assuming 1 g per cigarette) were calculated as a cumulative dose indicator and categorised into one of the three groups: >0–20, >20–40 and >40 pack-years. Additional variables used in the analysis were chronic obstructive pulmonary disease (COPD) (chronic bronchitis) and/or emphysema.

Data on all first-degree relatives were evaluated simultaneously using the following approach. First, conditional logistic regression was used to assess the risk of lung cancer depending on the numbers of affected relatives, a potential dose–response relation. The ORs were adjusted for the subject's sex, age, commune of residence, the cumulative exposure to smoky coal, smoking history, birth order and number of relatives. In addition, we tested the mean difference in response levels between probands and controls using the extended Mantel–Haenszel test statistic (Landis *et al*, 1998).

Second, an estimating equation-based technique based on a previous approach by Zhao and Le Marchand (1992) and Le Marchand *et al* (1996) was applied to account for intrafamilial phenotypic correlations. In this approach, the association between the relatives' and the subject's disease status is described via the logistic regression model 
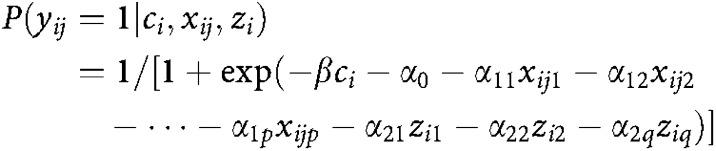
 where *y*_*ij*_ is a variable denoting the phenotype of person *j* related to study participant *i* (*y*_*ij*_=1 if diseased; else *y*_*ij*_=0), *c*_*i*_ is an indicator variable denoting the case–control status of the *i*th subject (c_*i*_=1 if study subject *i* is a case; else c_*i*_=0), *x*_*ij*_′=(*x*_*ij1*_, …, *x*_*ijp*_) is a vector of *p* covariates of the subject's relative *j* and *z*_*i*_′ =(*z*_*i*1_, …, *z*_*i*q_) is a vector of *q* covariates describing the participant, such as the matching variables. Subsequently, the regression parameters were obtained by solving a set of estimating equations. The overall familial aggregation was assessed by calculating the OR through the exponential function exp (*β*). The risk estimates were adjusted for the sex, age, commune of residence, the cumulation of smoky coal exposure, history of smoking, order of birth and generation (parent, sibling) of the relatives.

## RESULTS

Information was generated for 7206 persons. Of 851 probands identified, interviews were completed for 3697 of their first-degree relatives through 695 next-of-kin contacts. And of 839 spouses, interviews were conducted for 3310 of their relatives through 685 contacts. Information on complete two-generation pedigrees (nuclear families) was obtained for 740 (87%) proband families and 740 (88%) spouse families. An average of 3.4 and 3.6 interviews (contacts) was made to complete information on each of these proband and spouse families, respectively. The largest proportion of the contacts was siblings, followed closely by spouses. At the time of interviewing, about 82% of spouses (controls) were alive. Less than 18% of the contacts for cases and controls were adult offspring and surviving parents. The distributions of reported cancer in relatives by source of contacts were not significantly different between proband and spouse families. The remaining families of probands and spouses were excluded from further consideration because of inadequate information on names, commune of residence, and addresses to permit contact, nonresponses and insufficient responses from their immediate next of kin, or refusals to participate.

Death records of 85% of the 1848 dead relatives of probands and 1626 dead relatives of spouses were analysed. Information on cancer prevalence from death records did not corroborate that from interviews for 4.7% of relatives of probands and 4.3% of relatives of spouses (difference not significant at *P*>0.10). All lung cancer cases from probands' families and controls' families were confirmed and their data were analysed. In all, 13 proband families contained multiple probands, but each family was included in the data set only once (the earliest case as proband).

There were about 1.1 male probands to every female proband in our data set. The mean pedigree size was similar for both groups, 5.2 for the proband group and 5.1 for the spouses group. The differences in ages of surviving and deceased proband–spouse pairs were typically within the range of 0–5 years, respectively, although, on the average, no significant age differences were observed between the two groups of relatives (Table 1).

Of the 740 proband and 740 spouse families included in the final analyses, the distribution of cancers among their first-degree relatives is presented in Table 2. Only lung cancer will be discussed further in this paper. The crude OR estimate of a proband family having one first-degree relative affected by lung cancer was 2.82 times that of a spouse family (Table 2). Similarly, the OR for two and three lung cancers was 3.00 and 3.23 (*P*<0.05), respectively. For all other cancers, the excess risk was significant only in the case of families reporting at least two cancers (OR=2.37–4.57). Furthermore, Table 2 shows that the more the number of relatives affected, the higher the cancer (lung or other cancers) risk of other relatives unaffected in probands' families.

Furthermore, the distribution of lung cancer cases per family and corresponding risk estimates are presented in Table 3. While there are few families with multiple occurrences of lung cancer in relatives, families of probands are more often affected and show bigger clusters of affected family members (*P*-value=0.001).

When the distribution of lung cancer was analysed separately by age and sex groups, a significant difference between relatives of probands and relatives of spouses existed only in the age group over 40 years (at death or interview). The OR for lung cancer in male relatives of probands (>40 years old) was 3.1 (*P*<0.05) and for women 7.8 (*P*<0.05). Only 26.8% of the proband families reported no cancer compared with 48.7% of the spouse families. Cancers of the brain and nervous system, bone, larynx, oesophagus, stomach, kidney, bladder, ovary, endocrine glands and leukaemia lymphomas were more frequent (*P*<0.05) among the relatives of probands than of spouses.

No significant differences in the distribution of relatives' smoking status were noted between probands and spouses. Tobacco use, whether cigarettes, pipes or combination of tobacco types, was also similar for both groups. There were, however, 1.2 times more relatives of probands who had smoked more than two packs per day (*P*<0.05) and 1.1 times as many relatives of probands who had smoked an average of 50 or more packs-year (*P*<0.05). Both relatives of probands and spouses shared similar total duration of smoking. More than 96% of all smokers had smoked for 16 years or longer and about three-quarters for over 30 years. Nonsmoking female relatives of probands, however, showed more than 2.5-fold the risk for lung cancer of comparable relatives of spouses (Table 4). The proportions of relatives of probands and relatives of spouses who had been involved in smoky coal use did not differ appreciably.

The numbers of family members affected by lung cancer are listed in Table 4 by type of relative. Probands had 1632 siblings, of which 116 had a history of lung cancer. The number of controls' siblings was 1554 and, of these, 74 were affected. Joint analysis using the generalised estimating equations' technique revealed a steady increase in lung cancer risk among probands families after various adjustments (Table 4). In all cases, when the OR was determined by the logistic model, relatives of probands were at greater risk of lung cancer than the same relative of a spouse, adjusted age, sex, birth order, and commune of residence, COPD, smoking history and exposure to total smoky coal use. When the effects of all other variables were controlled for, however, the relationship (to proband or spouse) remained a significant determinant of cancer; the ORs for all female relatives and mothers of probands were 2.67 and 3.78 (*P*<0.01), respectively, and for all first-degree relatives and parents of probands 2.05 and 2.90 respectively, (*P*<0.01) compared with their spouse counterparts (Table 4).

## DISCUSSION

This study supports previously reported familial aggregation of lung cancer (Tokuhata and Lilienfeld, 1963; Ooi *et al*, 1986; Bromen *et al*, 2000; Wu *et al*, 2004). Using logistic regression, we found that this increased risk persisted after adjusting for age, sex, number of relatives, birth order, and commune of residence, COPD and history of smoking and smoky coal use exposures. *P* risk was elevated for those whose parent or siblings were affected by the disease. An overall familial aggregation is also indicated by the results of the generalised estimating equations' approach.

The crude risk for lung cancer among first-degree relatives of probands can be regarded as closely approximating to the true excess risk after accounting for any competing effects of age, sex, smoking and occupation; this risk was estimated to be 2.05 by logistic regression and far exceeds what might be expected by chance alone, that is, if a random sample of families had been obtained and was not inflated through the use of spurious controls (Haenszel, 1959). The 2.90-fold greater risk among parents of probands compared with those of spouses implies that a familial risk is detectable in different generations. When more than one proband was identified in a family, this was included only once in the analysis, thereby minimising the familial aggregation while still maintaining the independence of the families in the statistical analyses. The lack of differences in age, sex ratio, pedigree size, relationship types or mortality between proband and spouse families suggests that the two groups were well matched (see Table 1).

Our finding may be interpreted as supporting a genetic susceptibility to lung cancer. When we created a separate ‘environmental index’ for proband and spouse families by combining the regression coefficients for all variables other than the relationship variable, the resulting bivariate correlation coefficient was 0.62 (*P*<0.0001). Variations in the propensity of lung cancer developing in response to environmental factors (as explained by the nonrelationship components of the best regression model obtained) could not be statistically accounted for 62% of the time. Thus, the role of a putative genetic factor in cancer causation is evident here; otherwise, the response of proband families to environmental agent(s) would be expected to parallel closely that of spouse families. Moreover, the finding that cancers of the larynx, brain and nervous system, bone, endocrine glands, ovary, kidney, bladder, oesophagus, and stomach and leukemia lymphomas (as a group) were more prevalent among first-degree relatives of probands raises the possibility of a susceptibility to cancers in general or to a set of specific cancers.

The notion of a genetic contribution to lung cancer development derives support from several types of studies. First, the examination of host susceptibility markers in molecular epidemiologic and other studies has pointed to the role of polymorphisms in genes coding for phase I-activating (cytochrome *P*450 (*CYP*)*1A1, CYP2D6, CYP2E1*) and phase II-detoxifying (glutathione *S*-transferase (*GST*)*M1, GSTT1*) enzymes. More recently, these studies have begun to evaluate whether germline mutations and polymorphisms and methylation in oncogenes (*ras*) and tumour suppressor genes (*p53*, *p16*, *p15*) are potentially useful markers of genetic susceptibility (Sugimura *et al*, 1990; Kawajiri *et al*, 1993; Semenza and Weasel, 1997; Spivack *et al*, 1997; Esteller *et al*, 1999; Toyooka *et al*, 2003), although their findings have been inconsistent and controversial.

However, familial aggregation studies require cautious interpretation. With family members sharing lifestyle and other environment factors, it is difficult to obtain conclusive evidence about a disease having a genetic origin. Another frequent limitation is that a family history of a particular disease is often provided by the study subjects themselves without independent verification. However, a potential nondifferential misclassification of familial lung cancer would usually lead to an underestimation of the true risk and therefore could not explain the risk elevation.

More serious in this context is recall bias causing differential misclassification, but we do not believe that this has severely distorted our results for two reasons. First, we consider that lung cancer in a first-degree relative is severe enough to be remembered by both probands and controls without much difficulty. Second, we assessed this issue by asking subjects about multiple sclerosis in their relatives, a disease that is also severe but unrelated to lung cancer and found broadly similar numbers of relatives with multiple sclerosis in both probands and controls, even slightly more in the families of controls. Moreover, when comparing overall recall of diseases in family members, we found this slightly greater among controls than probands.

Overall, these findings support the idea that genetic susceptibility might act as an independent risk factor modifying the effect of exogenous risk factors, with smoky coal exposure and smoking being the most important.

## Figures and Tables

**Table 1:** Characteristics of families

**Family members and characteristic**	**Proband no. (%)**	**Mean age (years) of proband**	**Spouse no. (%)**	**Mean age (years) of spouse**
Families	740		740	
All relatives	3112		3034	
Male relatives	1736 (100.0)		1676 (100.0)	
Living	774 (45.0)	50.0	818 (49.0)	47.3
Dead	962 (55.0)	43.9	858 (51.0)	50.4
Female relatives	1376 (100.0)		1358 (100.0)	
Living	490 (36.0)	47.7	590 (43.0)	51.0
Dead	886 (64.0)	41.7	768 (57.0)	57.0
Parents	1480 (100.0)		1480 (100.0)	
Living^a^	130 (9.0)	67.8	202 (14.0)	68.0
Dead	1350 (91.0)	59.5	1278 (86.0)	60.1
Siblings	1632 (100.0)		1554 (100.0)	
Living	1134 (70.0)	45.1	1206 (78.0)	45.4
Dead	498 (30.0)	42.8	348 (22.0)	49.6

a *P*<0.01, between proportion of relatives in proband and spouse groups who were parents.

**Table 2:** Distribution of lung and other cancers in proband and spouse families

**Cancer**	**No. of affected persons in family^a^**	**Proband families**		**Spouse families**		**OR^b^**	**95% CI**
		**No.**	**%**	**No.**	**%**		
Lung cancer only	1	109	14.8	81	10.9	2.82**	1.95–4.82
	2	29	3.9	11	1.5	3.00*	1.15–5.97
	3	10	1.4	2	0.3	3.23*	1.05–15.30
	4	3	0.4	0	0.0	—	—
							
Cancer other than lung	1	81	10.9	63	8.5	1.62	0.93–2.97
	2	19	2.6	12	1.8	2.37*	0.98–3.46
	3	8	1.1	4	0.5	2.59**	1.32–5.37
	⩾4	2	0.3	1	0.1	4.57*	0.99–21.9

OR=odds ratio; CI=confidence interval.

* *P*<0.05.

** *P*<0.01.

a Excludes probands and spouses.

b Crude OR estimate of cancer for relatives of probands *vs* relatives of spouses.

**Table 3:** OR for lung cancer according to the number of first-degree relatives (parents, siblings) affected by lung cancer

**No. of relative affected**	**Probands**		**Controls**		**OR^a^**	**95% CI**	**OR^b^**	**95% CI**
	**No.**	**%**	**No.**	**%**				
0	589	79.5	646	87.3	1.00		1.00	
1	109	14.8	81	10.9	1.48*	1.07–2.03	1.41*	1.03–1.99
2	29	3.9	11	1.5	2.89**	1.37–6.21	2.69**	1.32–6.03
3	10	1.4	2	0.3	5.48*	1.13–36.4	5.40*	1.11–35.9
4	3	0.4	0	0.0	—	—	—	—
	740	100.0	740	100.0				
*P*-value	0.001							

OR=odds ratio; CI=confidence interval.

* *P*<0.05.

** *P*<0.01.

a OR, not adjusted.

b OR, adjusted for age, sex, size of the family, commune of residence, COPD, smoking and cumulation index of smoky coal exposure.

**Table 4:** Presence or absence of lung cancer in relatives of probands and relatives of spouses

**Relative**	**Lung cancer presence**	**Proband**	**Spouse**	**OR1^a^**	**95% CI**	**OR2^b^**	**95% CI**
Father	Yes	43	20	2.22*	1.26–3.95	2.17**	1.21–3.86
	No	697	720				
Mother	Yes	50	15	3.50*	1.89–6.58	3.78*	2.03–7.12
	No	690	725				
Parent	Yes	93	35	2.77*	1.83–4.19	2.90*	1.97–4.32
	No	1387	1445				
Brother	Yes	61	47	1.23	0.82–1.86	1.21	0.79–1.80
	No	935	889				
Sister	Yes	55	27	2.07*	1.26–3.42	2.23*	1.46–4.00
	No	581	591				
Sibling	Yes	116	74	1.53*	1.12–2.09	1.65**	1.19–2.18
	No	1516	1480				
Male^c^	Yes	104	67	1.53*	1.10–2.12	1.33**	1.02–2.02
	No	1632	1609				
Female	Yes	105	42	2.59*	1.77–3.80	2.67*	1.90–3.94
	No	1271	1316				
Total	Yes	209	109	1.93*	1.51–2.47	2.05*	1.68–2.53
	No	2903	2925				

OR=odds ratio; CI=confidence interval.

* *P*<0.01.

** *P*<0.05.

a OR1, estimate of lung cancer for relatives of probands *vs* relatives of spouses, not adjusted.

b OR2, estimate of lung cancer for relatives of probands *vs* relatives of spouses, adjusted for age, sex, birth order, size of the family, commune of residence, COPD, smoking and cumulation index of smoky coal exposure.

c Fathers and brothers.
